# Malakoplakia of the Pancreas with Simultaneous Colon Involvement: Case Report and Review of the Literature

**DOI:** 10.1155/2015/649136

**Published:** 2015-05-21

**Authors:** Surya Guha, Haiyan Liu

**Affiliations:** Geisinger Medical Center, 100 North Academy Avenue, Danville, PA 17821, USA

## Abstract

Although malakoplakia has been reported to occur at various body sites, pancreatic malakoplakia with simultaneous colonic involvement is very rare. Lesions of malakoplakia can masquerade as tumor masses leading to unwanted resections. Nevertheless, malakoplakia can occur in association with frank carcinomas, especially in the colon. By reporting a case of pancreatic malakoplakia diagnosed by fine needle aspiration cytology, this paper aims to describe cytologic features of malakoplakia and to further review findings from previous reported cases of pancreatic malakoplakia in the literature. With advances in minimally invasive, image-guided aspiration technologies, cytomorphological analyses can diagnose lesions of malakoplakia. This may help avoid surgeries that would have otherwise been carried out due to a misleading impression of tumor. However challenges remain as to the further management of patients diagnosed with this condition. More studies are necessary to determine the probability of malignancies arising in association with malakoplakia, in order to devise appropriate treatment protocols.

## 1. Introduction

A seemingly indolent disorder of immune function, malakoplakia, has been known to occur in various body sites, most commonly involving the urinary tract (in at least 70% of cases), with isolated reports of involvement of the colon, stomach, liver, pancreas, skin, and brain. On several occasions, malakoplakia presents as a “mass lesion” misleading clinicians toward a suspicion of a tumor. Conversely, malakoplakia has been shown to occur in organs otherwise harboring a malignancy (usually a carcinoma). In these instances, the malakoplakia occurs either as a response to the tumor or as an inciting agent. Other notable associations of malakoplakia include immune deficiency disorders, autoimmune conditions, organ transplantation (as a consequence of immunosuppression), and metabolic syndromes. Malakoplakias involving the pancreas are rare and may be diagnosed incidentally during elective cholecystectomy procedures [1]. Malakoplakias are infrequently diagnosed by cytological studies [2]. Diagnosis on cytology is further dependent upon the organ of involvement, which in turn determines the feasibility of performing fine needle aspirations. Reports of simultaneous or sequential organ involvement by malakoplakia are rare. We report a case of simultaneous involvement of the sigmoid colon and pancreas in a 59-year-old male with no known risk of immunodeficiency. Diagnosis of the sigmoid colon malakoplakia was performed on a surgical biopsy/polypectomy while the pancreatic involvement was diagnosed on fine needle aspiration. To our knowledge this is the first reported case of pancreatic malakoplakia to be diagnosed on cytology.

## 2. Case Presentation

A 59-year-old gentleman presented to our institution with anemia (hemoglobin: 9.4 g/dL) noted during preoperative evaluation for a cataract surgery. He was asymptomatic and had no history of immunodeficiency. His only previous history was a cholecystectomy performed 11 years prior to his current admission. He was noted to have a positive fecal occult blood test prompting colonoscopic evaluation revealing an 8 mm sessile polyp in the sigmoid colon which was resected. A second polyp, 3 mm in size, was identified within the ascending colon. No other large masses or mucosal ulcerations were identified. A few weeks later, the patient presented with abdominal pain and nausea, and a Computed Tomography (CT) scan of the abdomen was performed which revealed a vague mass like area within the pancreas. This was followed up with an endoscopic ultrasound which revealed a 33 × 32 mm mass with well-defined borders involving the pancreatic head. No cystic areas were present within the mass. No other abnormalities were detected in the remainder of the pancreas, bile duct, or liver. A fine needle aspiration of the pancreatic mass was performed.

During the next 3-4 weeks, the patient developed gradually worsening abdominal pain, fatigue, and weight loss. A repeat abdominopelvic imaging study revealed ascites and the pancreatic mass which had slightly enlarged. Cultures of the ascitic fluid grew* Escherichia coli* and hence antibiotic therapy was initiated. Impaired glucose tolerance was detected in the ensuing hospital stay. His general condition remained unimproved and he continued to experience reduction in weight and loss of appetite. Having counseled the patient on the possibility of a malignancy occurring in association with malakoplakias and in the absence of any other cause for his cachectic symptoms, the patient elected to undergo a pancreaticoduodenal resection (Whipple procedure). The patient reported improvement in his symptoms following surgery. Three months after surgery, he developed recurrent ascites with no additional imaging abnormalities detected.

## 3. Pathologic Findings

**Figure 1:**
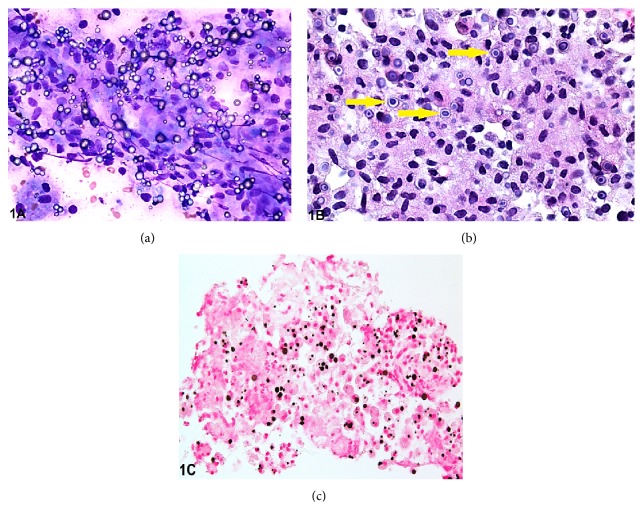
(a) Pancreatic fine needle aspirate showing many histiocytes with admixed lymphocytic tangles. Many refractile bodies with concentric laminations are identified (Diff-Quik, original magnification ×400). (b) Cellblock from pancreatic fine needle aspirate showing a granuloma containing many targetoid Michaelis-Gutmann bodies (arrows) (hematoxylin and eosin, original magnification ×400). (c) Von-Kossa stain highlighting the Michaelis-Gutmann bodies (original magnification ×400).

The fine needle aspirate obtained from the pancreatic head revealed abundant histiocytes and few admixed lymphocytes. Majority of the histiocytes were present in large sheets. On the Diff-Quik stained smears, the histiocytes were found to contain refractile bodies (Figure 1(a)). Occasional granulomas and focal aggregates of neutrophils along with scant necrotic debris were also present. The background contained few ductal and acinar cells. Fibrous stromal tissue was also identified. No dysplastic changes or overt features of malignancy were present. The cellblock revealed many granulomas containing distinctive targetoid bodies (Michaelis-Gutmann bodies) within the histiocytes (Figure 1(b)). A Von-Kossa stain performed on the cellblock material revealed dark-brown to black staining of the Michaelis-Gutmann bodies (Figure 1(c)). No organisms were identified by special stains (acid-fast stain, Grocott's methenamine silver stain). A CD68 immunoperoxidase stain highlighted the histiocytes.

**Figure 2:**
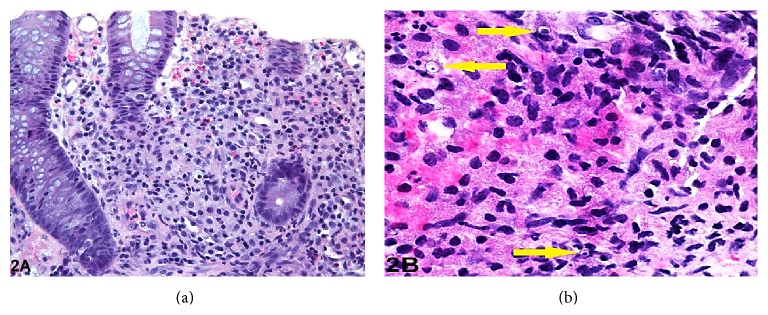
(a) Sigmoid colon biopsy showing lymphohistiocytic infiltration of lamina propria (hematoxylin and eosin stain, original magnification ×200). (b) Higher power view of sigmoid colon biopsy showing Michaelis-Gutmann bodies (arrows) (hematoxylin and eosin, original magnification ×400).

The sigmoid colon polyp showed lymphohistiocytic infiltration of the lamina propria and submucosa (Figure 2(a)). Few scattered Michaelis-Gutmann bodies were identified (Figure 2(b)). Occasional neutrophils and eosinophils were present. The colonic mucosal epithelium was unremarkable. The polyp resected from the ascending colon showed a tubular adenoma.

**Figure 3:**
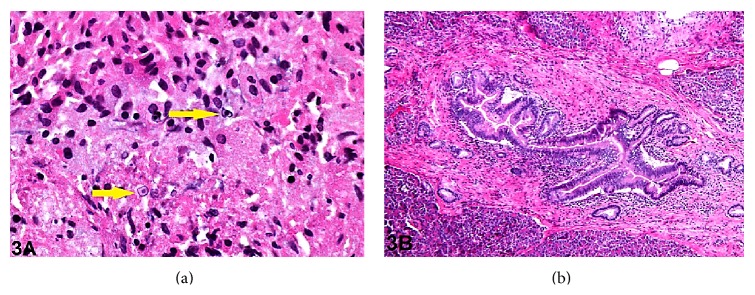
(a) Section from pancreatic resection showing histiocytes with Michaelis-Gutmann bodies (arrows) (hematoxylin and eosin, original magnification ×400). (b) Focus of pancreatic intraepithelial neoplasia, PanIN-2 (hematoxylin and eosin, original magnification ×600).

On gross examination of the Whipple specimen, the pancreatic parenchyma was indurated and mildly congested. No discrete mass was present. No mucosal abnormalities were visible within the distal stomach, duodenum, or the common bile duct. On light microscopy, sections from the pancreatic head revealed established chronic pancreatitis with many areas of lymphocytic inflammation, acinar and ductal atrophy, and fibrosis. Few scattered plasma cells were present. Additional areas of malakoplakia adjacent to atrophied pancreatic acini were identified (Figure 3(a)). Interestingly, focal low-intermediate grade pancreatic intraepithelial neoplasia (PanIN-2) was observed (Figure 3(b)). No evidence of high grade dysplasia or adenocarcinoma was identified in the pancreas or ampullary region after thorough histopathological examination. The duodenal mucosa and bile duct epithelia were unremarkable. The surgical resection margins were benign. Fifteen peripancreatic lymph nodes were examined and were found to be benign.

## 4. Discussion

In recognition of the original discoverers of this condition, malakoplakia is ubiquitously characterized by the presence of targetoid mineralized bodies known as Michaelis-Gutmann bodies. In fact a diagnosis of malakoplakia may be missed in the setting of a paucity of these characteristic bodies [3]. These represent undigested products from phagocytosed bacteria which, owing to their mineralization by calcium and iron, stain positively with the Von-Kossa and Prussian blue stains.* Escherichia coli* infection has been identified in many cases of malakoplakia involving the urinary tract, although other bacteria, viruses, and even protozoa have been implicated [4]. Malakoplakias are commonly observed to occur in immunocompromised patients. Why the great majority of immune deficient patients do not present with the lesions of malakoplakia remains unclear; specific errors in mechanisms involving the cyclic guanosine monophosphate pathway that regulates lysosomal function appear to play a determining role. Our patient was noted to have frank diabetes soon after his diagnosis of pancreatic malakoplakia. It is uncertain whether the diabetes was the causative factor for, or end result of, the pancreatic malakoplakia. Occasional reports of malakoplakia affecting immunocompetent individuals also exist in the literature. Systemic involvement by malakoplakia has been rarely reported. Our case may be the fourth in a series of case reports detailing systemic involvement by malakoplakia. Previous reports include a 49-year-old male who presented with retroperitoneal malakoplakia and subsequent bone involvement [3], a 6-week-old male with congenital immunodeficiency who presented with appendiceal and colonic malakoplakia, in addition to pancreatic involvement [5], and a 74-year-old male with pancreatic tail involvement by malakoplakia, along with spread to the peripancreatic lymph nodes [6].

The most common site of involvement of malakoplakia is the urinary tract. Malakoplakias involving the urinary bladder often present as white plaques noted on cystoscopic examination. The second most frequently involved site is the gastrointestinal tract, particularly the colon. Involvement of various other sites continues to be reported in the literature. As mentioned earlier, cases of malakoplakia involving the pancreas are relatively rare. A PubMed search revealed three publications reporting malakoplakia involving the pancreas. Zuk et al. [7] describe two additional cases of pancreatic malakoplakia in their review article. Relevant findings in these case reports are summarized in Table 1. Two patients [6, 7] presented with abdominal pain prompting further evaluation, while, in one patient, it was detected incidentally during elective cholecystectomy [1]. One of the patients described by Colby [3] was diagnosed with malakoplakia (secondary to* Corynebacterium equi* and* Klebsiella*) which presented as a retroperitoneal mass with additional involvement of the pancreatic tail and mesenteric root of the transverse colon. A year after antibiotic therapy he developed recurrent malakoplakia involving the right femur which also grew* Corynebacterium*, raising the possibility of immunodeficiency in the patient. Zuk et al. [7] described a patient with concurrent malakoplakia and moderately differentiated adenocarcinoma of the pancreas with widespread metastasis of the tumor and subsequent demise of the patient. Reports of malakoplakia involving lymph nodes have been described [6, 8] and it appears that diffuse lymph node involvement is usually more commonly seen in immunocompromised patients. The significance of lymph node involvement by malakoplakia is uncertain at present with some reports describing a poor prognostic implication [8], while in others [6] the patients have an uneventful postdiagnosis period.

Malakoplakia is notorious for mimicking a malignant neoplastic process. In fact, in a great majority of cases, the diagnosis first comes to light when a resection has been undertaken for the “malignant neoplasm.” Conversely malakoplakias have been reported to occur in association with adenocarcinomas including those of the colon and prostate [9–12]. Malakoplakia can pose significant challenges in staging of tumors as overstaging due to an overestimation of the size of the tumor may occur. Interestingly, malakoplakia is reported to occur at sites of prior radiation treatment which can be falsely Positron Emission Tomography (PET) positive, leading to an impression of tumor metastasis or recurrence [13].

No specific treatment protocols have been established in the management of malakoplakia. Although primarily considered a disorder of immune function, malakoplakia by itself is not particularly debilitating and patients diagnosed with lesions of malakoplakia generally do well. Fluoroquinolones, among other classes of antibiotics, have been utilized in the management of malakoplakia, especially in immunocompromised patients or those with systemic involvement, due to their ability to achieve therapeutic concentrations within histiocytes [14, 15]. Malakoplakia occurring in patients rendered immunocompromised by posttransplant immunosuppressive therapy may experience an improvement in general well-being by a trial of reduction in immunosuppression.

Malakoplakias continue to be reported to occur at unusual sites, such as the spleen, nasal cavity, and oropharynx [16–18]. The exact frequency of the association of malakoplakia with malignancy is not known at present and whether all cases of malakoplakia are to be definitively managed with surgery due to this risk is open for deliberation. Perhaps the greatest significance of this condition lies in the fact that it can be frequently mistaken for malignancy. Hence minimally invasive techniques such as fine needle aspiration or biopsies wherever possible may be undertaken to examine the true nature of these mimickers of malignancy.

## Figures and Tables

**Table 1:** Summary of features of pancreatic malakoplakia in the literature.

Reference article number	Age (in years) and gender of patient	Clinical presentation	Additional findings	Clinical progression
[1]	75, male	Pancreatic mass detected during elective cholecystectomy	No other organ involvement	No complications after excision of pancreatic mass

[3]	49, male	Weight loss and anemia	Retroperitoneal malakoplakia	Bone recurrence of malakoplakia

[5]	6 weeks, male	Immunodeficiency	Adrenal and colonic involvement by malakoplakia	Expired due to military tuberculosis

[6]	74, male	Fever and abdominal pain, pancreatic tail mass	Peripancreatic lymph nodes involved by malakoplakia	Well, 13 months after distal splenopancreatectomy

[7]	45, female	Recurrent abdominal pain	Moderately differentiated pancreatic ductal adenocarcinoma with metastasis	Expired 10 months after Whipple procedure
